# Enhanced simulations of whole-brain dynamics using hybrid resting-state structural connectomes

**DOI:** 10.3389/fncom.2023.1295395

**Published:** 2023-12-19

**Authors:** Thanos Manos, Sandra Diaz-Pier, Igor Fortel, Ira Driscoll, Liang Zhan, Alex Leow

**Affiliations:** ^1^ETIS, ENSEA, CNRS, UMR8051, CY Cergy-Paris University, Cergy, France; ^2^Laboratoire de Physique Théorique et Modélisation, UMR 8089, CNRS, Cergy-Pontoise, CY Cergy Paris Université, Cergy, France; ^3^Simulation and Data Lab Neuroscience, Institute for Advanced Simulation, Jülich Supercomputing Centre (JSC), JARA, Forschungszentrum Jülich GmbH, Jülich, Germany; ^4^Department of Biomedical Engineering, University of Illinois at Chicago, Chicago, IL, United States; ^5^Department of Psychology, University of Wisconsin-Milwaukee, Milwaukee, WI, United States; ^6^Department of Electrical and Computer Engineering, University of Pittsburgh, Pittsburgh, PA, United States; ^7^Department of Psychiatry, University of Illinois at Chicago, Chicago, IL, United States

**Keywords:** whole brain dynamics, resting-state brain dynamics, neuroimaging data, functional connectivity, resting-state informed structural connectome, Alzheimer's disease

## Abstract

The human brain, composed of billions of neurons and synaptic connections, is an intricate network coordinating a sophisticated balance of excitatory and inhibitory activities between brain regions. The dynamical balance between excitation and inhibition is vital for adjusting neural input/output relationships in cortical networks and regulating the dynamic range of their responses to stimuli. To infer this balance using connectomics, we recently introduced a computational framework based on the Ising model, which was first developed to explain phase transitions in ferromagnets, and proposed a novel hybrid resting-state structural connectome (rsSC). Here, we show that a generative model based on the Kuramoto phase oscillator can be used to simulate static and dynamic functional connectomes (FC) with rsSC as the coupling weight coefficients, such that the simulated FC aligns well with the observed FC when compared with that simulated traditional structural connectome.

## 1 Introduction

The human brain is a complex neural network that self-organizes into different emergent states, which are crucial for its functions. Such states include spatiotemporal patterns of neural synchronization associated with cognitive processes (Bansal et al., 2019). Brain regions can be modeled as dynamically interacting nodes in a functional network on a 3D space (functional brain networks), which was coupled in a complex manner driven by the structure of these networks. Over the past years, interdisciplinary approaches, using concepts from non-linear dynamics, physics, biology, and medicine to name a few, allowed us to understand in more depth how the human brain functions and how certain brain disorders and their underlying mechanisms can be further studied using mathematical models. It is feasible to ameliorate even more the predictive performance of such models, since a vast amount of neuroimaging data e.g., electroencephalography (EEG), magnetoencephalography (MEG), and functional magnetic resonance blood-oxygen-level-dependent (BOLD) functional magnetic resonance imaging (fMRI) became available in the last two to three decades. Such data may provide information not only for healthy or pathological brain activity but can also be used to fingerprint functional connectomes by identifying individuals using brain connectivity patterns (Finn et al., 2015).

Together with extensive experimental study, mathematical/computational modeling of the whole brain dynamics has been an active research topic for years (Deco et al., 2008; Sanz-Leon et al., 2015; Jirsa et al., 2017; Murray et al., 2018; Young, 2020). In such a setting, one can model populations of neurons as nodes in a graph structure. Then, one can obtain information about relative connection weights (coupling strength) and communication lag (delay) between different nodes by diffusion-weighted magnetic resonance imaging (dwMRI) techniques (Ghosh et al., 2008; Hagmann et al., 2010; Deco et al., 2011). This is termed as the structural connectivity (SC) of the network, and it is, in general, subject-dependent with a certain degree of variability (gender/age/healthy vs. diseased). Furthermore, statistical analysis of BOLD time series inferred from fMRI can provide the functional relationships between different brain regions. It is usually calculated as the Pearson correlation coefficient of the activity between regions and results in the empirical functional connectivity (FC) matrix per brain recording and subject (Sporns et al., 2005; Horn et al., 2014).

By working *in silico*, one can seek for model parameters that are able to produce simulated time series and global dynamics that fairly resemble the empirical ones. One way in achieving that is to tune selected parameters which optimize the similarity between empirical FC and simulated FC (Cabral et al., 2011; Deco and Jirsa, 2012). Hence, these parameters can serve as dynamical biomarkers and predictors of different brain states and behavioral modes [see Popovych et al. (2019) for a recent review]. Along this direction, the virtual epileptic patient has been recently proposed, where medical-treatment approaches using personalized mathematical models for epileptic patients have been illustrated (Jirsa et al., 2017). Furthermore, the choice of the brain atlases, (i.e., the mapping of the different regions of interest (ROIs) based on functional or anatomical criteria using different parcellations) can affect the quality of model performance and its level of agreement with the empirical data [see Popovych et al. (2021) and references therein for more details].

In recent years, substantial research efforts have been directed toward understanding the brain (large-scale activity) using resting state fMRI (rs-fMRI), employing sophisticated mathematical and statistical tools to investigate the FC from rs-fMRI data (Biswal et al., 1997). So far, the mainstream approach is to consider SC to be static and the FC to be dynamic. However, this is not necessarily the case as the white matter tracts can be in use or engaged when the brain is performing certain tasks but inactive or disengaged during other tasks and hence not static. An altered and more sophisticated “functional connectivity-informed structural connectivity” has been introduced by Ajilore et al. (2013), employing information from fMRI to infer the underlying pattern of white matter engagement specific to the state of the brain. The resulting connectome, the so-called resting-state informed structural connectome (rsSC), encodes the structural network that underlies and facilitates the observed rs-fMRI correlation connectome which is able to detect altered rsSC community structure in diseased subjects relative to controls. In the original set up, there is no “directionality” inferred, i.e., whether the white matter tract of interest is of “excitatory” vs. “inhibitory” nature.

However, understanding the dynamical balance between excitation and inhibition, a concept termed E-I balance is vital for adjusting neural input/output relationships in cortical networks and regulating the dynamic range of their responses to stimuli (Kinouchi and Copelli, 2006) such that information capacity and transfer are maximized (Shew et al., 2011). This is the central thesis of the criticality hypothesis (Beggs and Plenz, 2003; Muñoz, 2018), i.e., that brain activity self-organize into a critical state (Wilting and Priesemann, 2019), a unique configuration likened to a phase transition in physical systems where a dynamical system transitions from order (balanced excitation-inhibition) to disorder (disrupted excitation-inhibition balance) (Sornette, 2004; Cocchi et al., 2017; Hahn et al., 2017; Tagliazucchi, 2017). Indeed, evidence supporting that the brain is operating near criticality has been reported in studies examining neuronal signaling (Beggs and Plenz, 2003; Shew et al., 2009; Hahn et al., 2017) and BOLD fMRI signals (Tagliazucchi et al., 2012; Haimovici et al., 2013; Lombardi et al., 2017; Rabuffo et al., 2021).

To incorporate co-activation (excitatory) or silencing (inhibitory) effects into our hybrid rsSC framework that would allow us to infer the E-I balance of the brain, in the study by Fortel et al. (2019), we then introduced an improved framework based on the Ising model representation of the brain as a dynamical system, wherein self-organized patterns are formed through the spontaneous fluctuations of random spins. This Ising spin-glass model has been previously used to successfully characterize complex microscale dynamics (Tkacik et al., 2015; Kadirvelu et al., 2017) and macroscale interactions (Schneidman et al., 2006; Marinazzo et al., 2014; Ezaki et al., 2017; Nghiem et al., 2018; Niu et al., 2019; Nuzzi et al., 2020) of the human brain and to accurately represent spatiotemporal co-activations in neuronal spike trains (Schneidman et al., 2006; Shlens et al., 2006; Roudi et al., 2009) and patterns of BOLD activity (Watanabe et al., 2013; Ashourvan et al., 2017; Cocco et al., 2017; Ezaki et al., 2020).

In this study, we use The Virtual Brain (TVB, Sanz-Leon et al., 2015), a whole-brain simulation platform part of the EBRAINS infrastructure (https://ebrains.eu/), to investigate the potential benefits in employing rsSC instead of the traditional SC for simulating whole-brain dynamical activity. For example, one major limitation of using traditional SC with certain dynamical models, such as the Kuramoto phase oscillators (Kuramoto, 2003) and the generic limit-cycle oscillators (Kuznetsov, 1998), to model the mean neural activity of each node using traditional SC connectomes, is that the resulting signals from different ROIs do not adequately produce negative correlations obtained in the empirical ones, even in the presence of delays in the system (Popovych et al., 2021). Here, we show that using rsSC such as dynamical systems succeed to produce simulated signals with both positive and negative correlations which sufficiently follow the trends of the empirical ones.

## 2 Methods and materials

### 2.1 Empirical data and signed resting state structural connectome

The structural and functional connectivity (resting state) for 38 cognitively normal APOE ε4 allele carriers with the detailed information on the imaging and processing steps can be found in the study by Korthauer et al. (2018). The algorithm to obtain rsSC can be found in the study by Fortel et al. (2019, 2020); Tang et al. (2021); Fortel et al. (2022), while in the Supplementary material, we provide a concise description with details on its implementation.

### 2.2 Models and simulated data

To produce simulated fMRI time series in the given connectomes, we employ the Kuramoto phase oscillator model (Kuramoto, 2003; Lee and Frangou, 2017; Popovych et al., 2021):


(1) $$\begin{array}{l} {\overset{\cdot }{\theta }}_{i}\left(t\right)=2\pi f_{i}+\frac{K}{N}\overset{N}{\underset{j=1}{\sum }}c_{ij}\sin\left[\theta_{j}\left(t-\tau_{ij}\right)-\theta_{i}\left(t\right)\right],\;i=1,2,...,N \end{array}$$

where θ_*i*_ are the phases, *N* is the number of oscillators, *f*_*j*_ are the natural frequencies (Hz), *c*_*ij*_ and τ_*ij*_ (ms) represent the individual coupling weight and propagation delay in the coupling, respectively, from oscillator *j* to oscillator *i*, while *K* is the global coupling parameter. The time *t* in the model and delay in coupling term are measured in ms.

For each individual subject, we produced a “personalized” model Equation (1) to simulate dynamics of the network and calculate time series. To this end, two cases of connectivity matrices were compared: (i) in the first one, the *c*_*ij*_ values are defined by simply counting the number of streamlines connecting regions *i* and *j* normalized to 1 and with zero diagonal (i.e., define *c*_*ij*_ as a normalized version of the empirical tractography-derived SC or eSC), leading to only excitatory interactions between ROIs; and (ii) in the second one, the *c*_*ij*_ values are assigned by the corresponding entries of the hybrid rsSC connectomes, leading to both excitatory and inhibitory interactions between ROIs. The delays τ_*ij*_ were calculated as τ_*ij*_ = *L*_*ij*_/*V*, where *L*_*ij*_ (mm) is the average tract (path) length of the streamlines connecting regions *i* and *j*, and *V* (m/s) is an average velocity of signal propagation. In this particular dataset, the exact path lengths are not available; hence we used the euclidean distance between nodes in the Desikan atlas (Desikan et al., 2006) as proxies. The euclidean distance has been used in the literature in the construction of structural networks (Ercsey-Ravasz et al., 2013) and found to closely follow the trends obtained by anatomical tract-tracing studies. Furthermore in the study by Deco et al. (2021), the authors showed that such networks also strongly correlate with MRI tractography-based networks. The matrix **L** = *L*_*ij*_ can thus be used to calculate the delays τ_*ij*_ in the coupling, which can be expressed as τ_*ij*_ = τ·*L*_*ij*_/〈*L*_*ij*_〉, where τ = 〈*L*_*ij*_〉/*V* is the global (or mean) delay. In Equation (1), the self-connections were excluded by setting the diagonal elements in the matrices eSC/rsSC and **L** to zero (i.e., *c*_*ii*_ = *L*_*ii*_ = 0, respectively).

**Figure 1:**
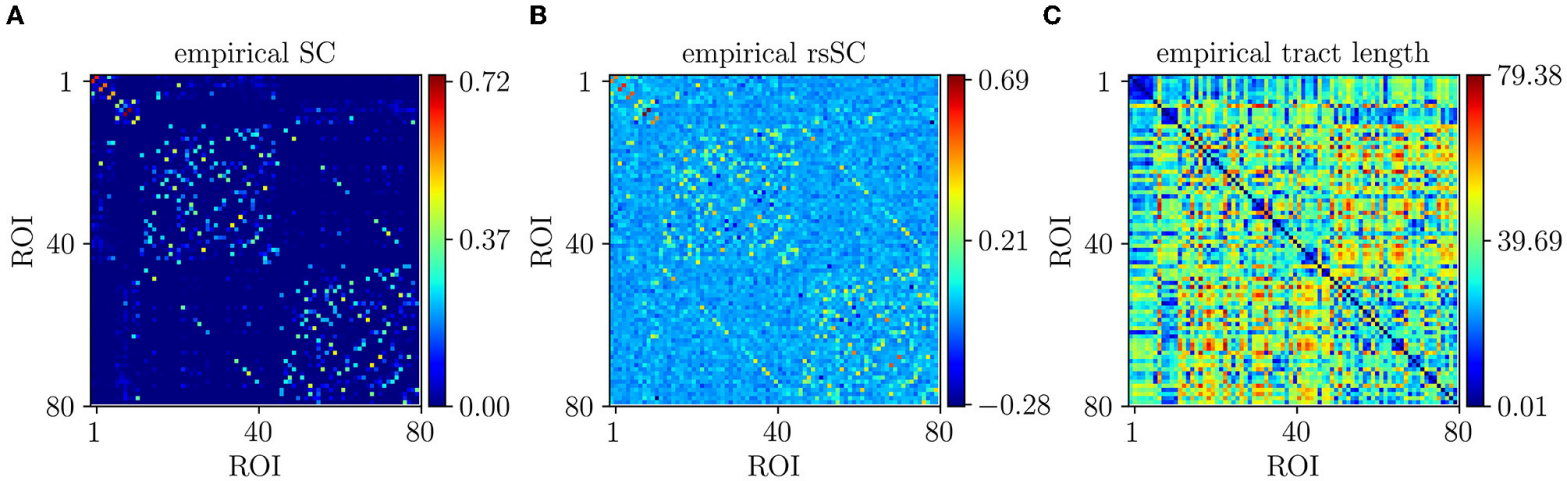
Empirical connectivity matrices example (non-carrier subject). **(A)** Weights of SC matrix. **(B)** Hybrid rsSC matrix. **(C)** Tract length (mm) matrix **L** based on the euclidean distance of the nodes on the Desikan atlas (same for all simulations and subjects).

In Figure 1A, we show the empirical SC matrix (weights of the node-to- node connections) for a Non-Carrier subject from the dataset with 80 nodes (ROIs). Figure 1B shows the corresponding SC and subject rsSC matrix calculated as described earlier. Notably, the hybrid rsSC contains negative entry values as opposed to SC one that is restricted to having only positive values. Figure 1C) shows the tract length **L** matrix (in mm) that we used for the simulation of all subjects in the absence of the actual measured ones from a neuroimaging prepossessing pipeline. The phases in our model (Equation 1) were initialized randomly. We set the intrinsic frequencies to be uniformly distributed with mean = 60 Hz and SD = 1 Hz, corresponding to oscillations within the gamma frequency range [see Cabral et al. (2011); Mess et al. (2014); Váča et al. (2015); Lee and Frangou (2017) for more details and motivation], as gamma local field potential (LFP) power is coupled to the BOLD fMRI signal and is considered representative of the overall neuronal activity (Niessing et al., 2005; Nir et al., 2007; Miller et al., 2009; Schölvinck et al., 2010)].

For our simulations, we used a TVB tailored version for the Kuramoto model and we made adjustments for efficient parallelization on CPUs using MPI on the supercomputer JUSUF located at the Jülich Supercomputing Center. Our model generates time series which correspond initially to electrical activity (fast oscillations) for each node, i.e., we register the observable *x*_*i*_ = sin(θ_*i*_) for each brain region. Then, in order to estimate the simulated BOLD signal, we use the built-in tool of TVB to calculate the hemodynamic response function kernel (i.e., “fMRI activity”) associated with a given neural activity time series, also known as the Balloon-Windkessel model (Friston et al., 2003). Our simulations ran for 500 s. The first 20 s were discarded to remove transient effects, resulting in *T* = 480 s (8 min), i.e., a time interval identical to the time-length of the empirical fMRI signals. We set the time-step at 0.1 ms, and we integrated the system with an Euler scheme. In this particular study, we did not consider the presence of noise.

## 3 Results

We numerically simulate BOLD time series varying two model parameters, namely, the global coupling strength *K* and the delay τ in Equation (1), with respective ranges *K* ∈ [1, 75] and τ ∈ [1, 33], resulting in a 32 × 32 grid. For each pair of parameters, we begin by producing the matrix of the simulated FC (sFC). The latter is measured by the Pearson Correlation Coefficient (CC) between the simulated BOLD signals *x*_*i*_, *i* = 1, 2, …, *N* from different ROIs [also referred to as Static Functional Connectivity in the literature, see Cabral et al. (2017)] as follows


(2) $$\begin{array}{l} {\text{CC}}_{\text{BOLD}}=\text{corr}\left(x_{i},x_{j}\right). \end{array}$$

Then, we compare each sFC with the eFC ones using the Pearson Correlation Coefficient; however, this time we calculate it for the two respective matrices (upper triangular parts) as follows:


(3) $$\begin{array}{l} {\text{CC}}_{\text{FC}}=\text{corr(sFC,eFC)}. \end{array}$$

The optimal match between sFC and eFC in the parameter space is acquired for (*K*, τ)-values where CC_FC_ becomes maximal [see also Popovych et al. (2021) and references therein for more details and motivation].

**Figure 2:**
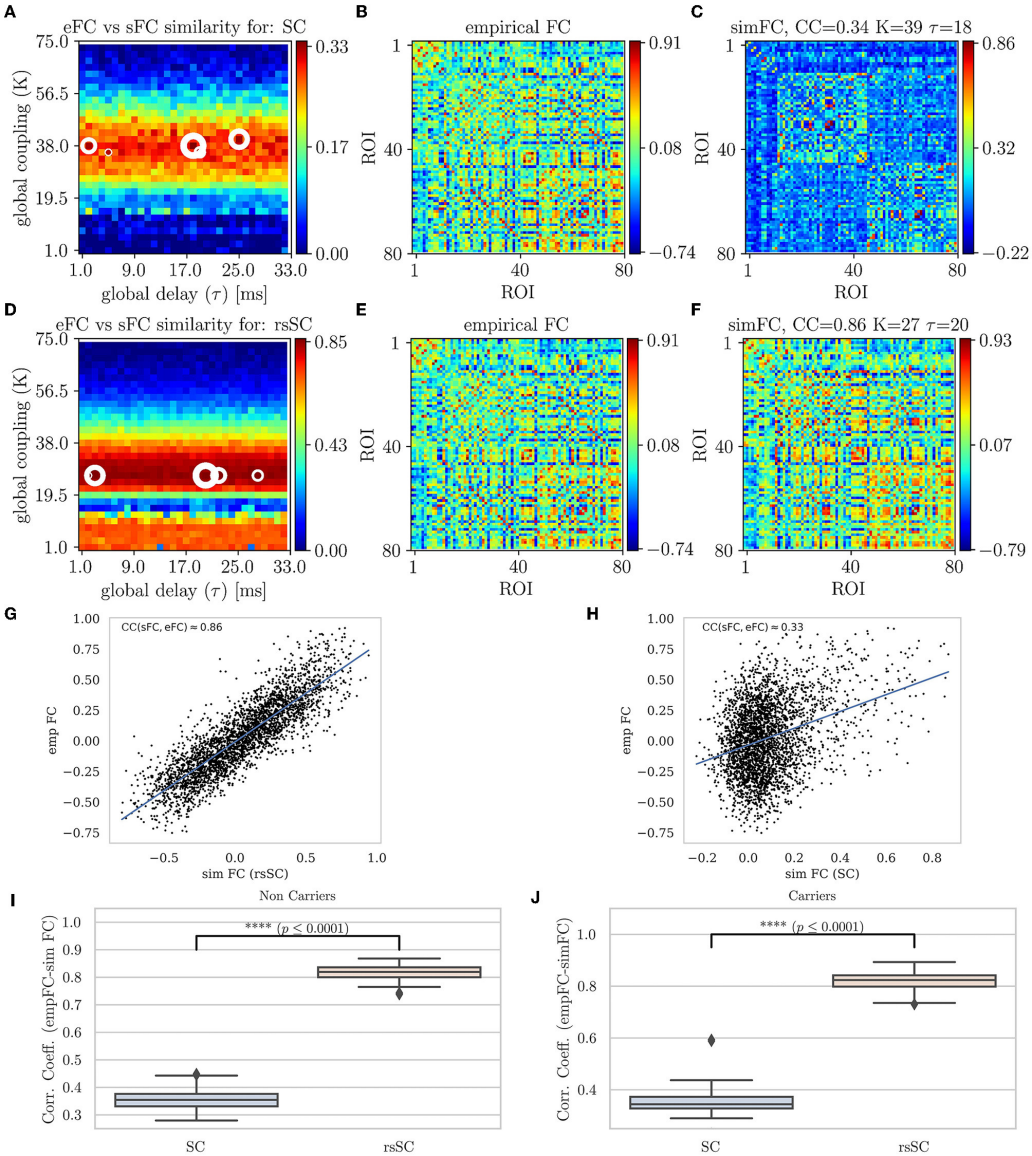
Parameter sweep exploration, correlation & Statistical analysis for eFC vs. sFC example. First row (using the respective subject's standard SC matrix to define the weights in model Equation (1): **(A)** The colormap depicts the CC_FC_ = corr(sFC, eFC) for the parameters (*K*, τ). The 5 circles on the red regions indicate the highest correlations found (larger circles' sizes correspond to larger CC_FC_ values). **(B)** The eFC calculated from the empirical BOLD signal. **(C)** The sFC matrix with the larger CC_FC_. Second row (using the respective subject's hybrid rsSC matrix to define the weights in model Equation (1). **(D)** The respective colormap for CC_FC_ = corr(sFC, eFC). **(E)** The eFC calculated from the empirical BOLD signal [same as **(B)**]. **(F)** The sFC matrix with the larger CC_FC_. Note the ranges for the two colorbars in **(A, D)** are kept intact (i.e., no scaling) for visualization purposes. **(G, H)** Scatterplots between empirical (*y*−axis) and optimal simulated BOLD correlations (*x*−axis) aggregated across all entries in the corresponding FC matrices, i.e., **(B, C)**. **(G)** eFC vs. sFC using rsSC, **(H)** eFC vs. sFC using standard SC. Both panels refer to same subject (blue lines indicate the linear regression model). **(I, J)** boxplots for the non-carriers and carriers dataset (38 subjects per type) correlation coefficients between eFC and sFC using SC and rsSC matrices for the simulated time series respectively. For each subject we considered the 5 maximum values (see circles in **(A, D)**). The difference in the respective mean values of the two datasets is statistically significant measured by the t-test with very small *p*−value (*p* ≤ 0.0001) for both non-carriers and carriers sets.

In Figure 2, we present the first main result, namely, the superiority of hybrid rsSC over standard SC matrices in generating simulated BOLD time series with models such as Equation (1), which better approximate the empirical BOLD signals (shown here for one example healthy subject). The upper row refers to simulations performed using the standard SC matrix of the respective subject to define the coupling weights in the Kuramoto model. Figure 2A shows the parameter sweep exploration (PSE) for eFC vs. sFC and for the parameters (*K*, τ) and measured as CC_FC_ = corr(sFC, eFC). The five white circles on the red regions indicate the highest correlations (sizes of larger circles correspond to larger CC_FC_ values). In Figure 2B, we present the eFC calculated from the empirical BOLD signal while in Figure 2C the sFC matrix with the larger cc_FC_. We can observe that sFC did not capture adequately the negative correlations that are present in eFC (compareing the minimum values in the barplots of Figures 2B, C).

In the second row of Figures 2D–F, we perform similar simulations; however, we now use the hybrid rsSC matrix of the respective subject to define the coupling weights in the Kuramoto model. Notably, the significant improvement in the maximum value of the CC_FC_≈0.86 compared with the one found when using the standard SC matrix (CC_FC_≈0.33). Notably, also the better agreement is observed between the two FC matrices (empirical (E) and simulated (F)) and how better the sFC captures both positive and negative correlations (indicated by the range of the respective colorbars). We should stress that we did not opt to use the same range for the two colorbars in Figures 2A, D, as in this way it would be difficult to visually identify the PSE region in (A), depicting the optimal parameter values.

The respective scatterplots and CC values between empirical and optimal simulated FC matrices are presented in the third row of Figure 2 using rsSC (G) and standard SC (H) matrices. Here, we plot the empirical (*y*-axis) against the optimal simulated BOLD correlations (*x*-axis) aggregated across all entries in the corresponding FC matrices; thus, a perfect match between the two would place all the points along the line *x* = *y*. The higher CC_FC_(sFC, eFC) value (using hybrid rsSC matrices) is well reflected by a rather clear linear trend in the distribution of the points (Figure 2A). On the other hand, only a relatively weak linear trend is obtained using standard SC matrices (Figure 2B). Both panels refer to the same subject presented in Figure 2, with the lines indicating the corresponding linear fit in each case.

In the forth row of Figure 2, we present a statistical analysis for all subjects per category, i.e., 38 non-carriers (I) and 38 carriers (J). For each subject, we considered the five maximum values of correlation coefficients between eFC and sFC using SC and rsSC matrices for the simulated time series, respectively, (circles in (A) and (D)) and produced boxplots. We then used the t-test to measure the difference in the mean Pearson correlation value of each group (carriers or non-carriers) between the empirical FC vs. simulated FC (5 optimal cases) when using standard SC and rsSC ones.. The difference in the respective mean values of the two datasets is found to be statistically significant with very small *p*-value (*p* ≤ 0.0001) for both non-carriers. The respective BOLD time series (empirical, optimal simulated using SC and rsSC respectively) from this example can be found in Supplementary Material (Supplementary Figure S1). The separation of the two groups is indeed biologically important given the already demonstrated differences in E-I dynamics (Fortel et al., 2019, 2020, 2022, 2023). As this current study leveraged one of the several datasets that we previously used to demonstrate sex-by-ε4 hyperexcitation, by showing that the model fits between the two groups are equally optimal we further establish that differences in E-I dynamics are not an artifact secondary to differences in model fit.

**Figure 3:**
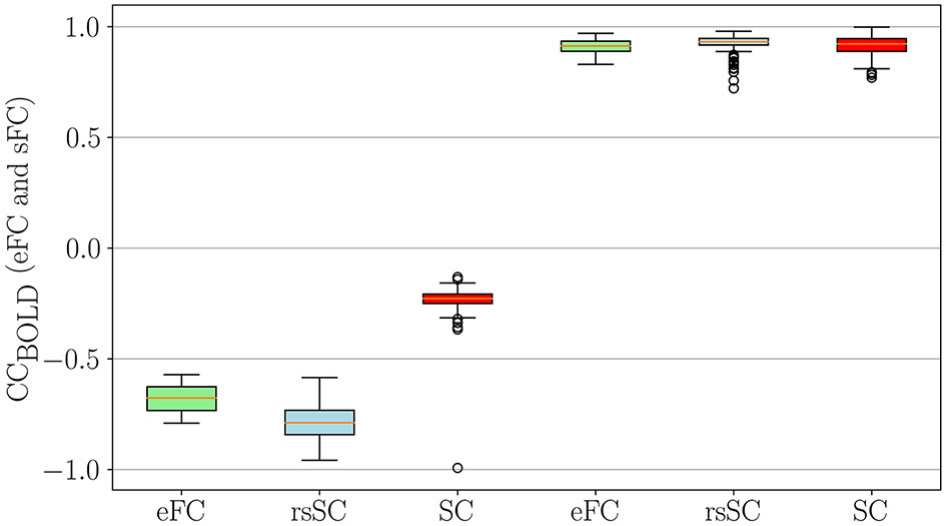
Statistical Analysis for negative and positive correlations in empirical and simulated FCs. Boxplots of the correlation coefficients for eFC and sFC obtained by using either SC or rsSC as the coupling coefficient matrices (non-carriers group) in the simulations.. For each subject we considered the parameters (*K*, τ) which correspond to the 5 maximum values that optimize the similarity between eFC and sFC matrices (indicated with circles in Figures 2 A, D)). The 3 leftmost boxplots indicate the negative correlations in the actual eFC (light green), simulated using rsSC (light blue) and using standard structural connectome SC (red). Similarly, the 3 rightmost boxplots compare the maximum (positive) correlation values. Similar results were also obtained for the carriers, see Supplementary Figure S2.

Next, we conducted a statistical analysis of negative and positive correlations in empirical (eFC) and simulated (sFC) functional connectomes (Figure 3). We used boxplots to visualize correlation coefficients for eFC and sFC matrices informed by structural connectivity (SC) or resting-state SC (rsSC) for the non-carrier group. We considered sFC matrices produced by the five parameters (*K*, τ) that maximized eFC and sFC similarity per subject (marked with circles in Figures 2A, D). The leftmost three boxplots show minimum (negative) correlations in actual eFC (light green), which was simulated using rsSC (light blue) and using standard SC (red), respectively, while the rightmost three boxplots show maximum (positive) correlation values.

The simulated functional connectomes (sFC), generated using both rsSC (used here for the first time in simulating BOLD time series) and standard SC, sufficiently recovered the positive correlations observed in the empirical BOLD signals. However, the sFC derived from SC (left red boxplots) do not correctly recover the negative correlations present in the empirical data (left light green boxplots). In contrast, the BOLD signals generated with rsSC (left light blue boxplots) exhibit negative correlations much closer to those observed in the empirical data. This consistent trend holds true for both non-carrier and carrier datasets (see Supplementary Figure S2). A more detailed analysis on the role of positive and negative coupling coefficients in rsSC vs. SC connectommes in simulating high-fidelity fMRI correlations with a dynamical system can be found in the discussion and figures of Supplementary material.

Next, to further explore the advantage in using the hybrid rsSC matrices beyond Static Functional Connectivity matrices, we sought out to perform a similar PSE analysis for Dynamic Functional Connectivity, which allows us to capture switching trends in the resting state activity. To this end, we calculate the Phase Coherence Connectivity [see e.g., Cabral et al. (2017); Hancock et al. (2022) and references therein], which does not suffer from time-window length effects such as other similar techniques based on calculating successive FC(*t*) matrices using a sliding-window [see discussion in Cabral et al. (2017); Hancock et al. (2022)]. Hence, we use BOLD Phase Coherence Connectivity to measure time-resolved dynamic FC matrices (dFC), with size *N* × *N* × *T*, where *N* refers to the number of ROIs and *T* = 236 refers to the total number of recording frames. Then, we begin by estimating the phases from the BOLD time series (empirical and simulated) for all ROIs *i* (θ(*i, t*)) applying a Hilbert transform and we bandpass filter the parcellated fMRI time-series within 0.01 − 0.1Hz [see e.g., Popovych et al. (2021) and references therein] using a discrete Fourier transform computed with a fast Fourier transform. Then, the phase coherence between brain areas *i* and *j* at time *t*, dFC(*i, j, t*) is defined as follows:


(4) $$\begin{array}{l} \text{dFC}\left(i,j,t\right)=\cos\left(\theta \left(i,t\right)-\theta \left(j,t\right)\right). \end{array}$$

When two ROIs have temporarily aligned BOLD signals their respective dFC(*i, j, t*)≈1 while their BOLD signals are orthogonal dFC(*i, j, t*)≈0. Notably, the matrix dFC serves as the foundation of Leading Eigenvector Dynamic Analysis (LEiDA) which has been used to detect subtle FC patterns that distinguish between healthy and diseased BOLD signals (Cabral et al., 2017; Hancock et al., 2022).

**Figure 4:**
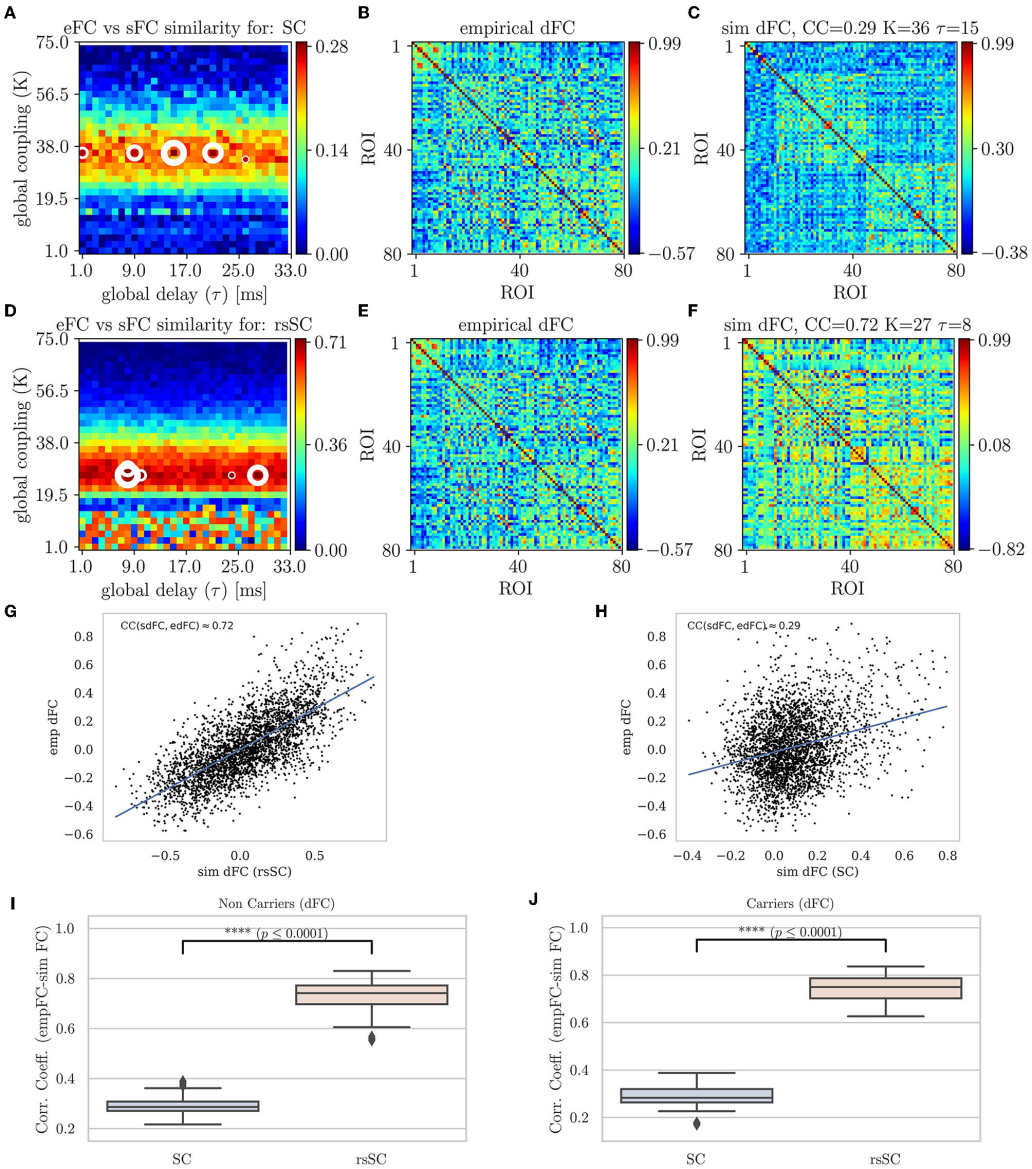
Parameter sweep exploration for phase coherence connectivity (edFC vs. sdFC). First row (using the respective subject's standard SC matrix to define the weights in model Equation (1): **(A)** The colormap depicts the CC_dFC_ = corr(sdFC, edFC) for the parameters (*K*, τ). **(B)** The edFC calculated from the empirical BOLD signal. **(C)** The sFC matrix with the larger CC_dFC_. Second row (using the respective subject's hybrid rsSC matrix to define the weights in the model. **(D)** The respective colormap for CC_dFC_ = corr(sdFC, edFC). **(E)** The edFC calculated from the empirical BOLD signal (same as **(B)**). **(E)** The sdFC matrix with the larger CC_dFC_. Note the different ranges in the respective colorbars of **(B–F)** capturing the different temporal alignment of the phase of the respective BOLD signals. See text for more details. **(G, H)** Scatterplots between empirical (*y*−axis) and optimal simulated dFC correlations (*x*−axis) aggregated across all entries in the corresponding dFC matrices, i.e., **(B, C)**. **(G)** edFC vs. sdFC using rsSC **(H)** edFC vs. sdFC using standard SC. Both panels refer to same subject (blue lines indicate the linear regression model). **(I, J)** Boxplots for the non-carriers **(I)** and carriers **(J)** dataset (38 subjects per type) correlation coefficients between edFC and sdFC using SC and rsSC matrices for the simulated time series respectively. For each subject we considered the 5 maximum values (see circles in **(A, D)**. The difference in the respective mean values of the two datasets is statistically significant measured by the t-test with very small *p*−value (*p* ≤ 0.0001) for both non-carriers and carriers sets.

In Figure 4, we present the Phase Coherence Connectivity PSE for edFC vs. sdFC (in a similar way as in Figure 2). However, we now compare each simulated mean dFC (sdFC) calculated by Equation (5) with the empirical (edFC) ones using the Pearson Correlation Coefficient from the upper triangular section of the two respective matrices as follows:


(5) $$\begin{array}{l} {\text{CC}}_{\text{dFC}}=\text{corr(sdFC,edFC)} \end{array}$$

The optimal match between sdFC and edFC in the parameter space is acquired for (*K*, τ)-values where CC_dFC_ becomes maximal (in Figures 4A, D, we indicate five maximum values with white circles). The upper row shows the results when we use the standard SC matrix of the respective subject to define the weights in model Equation (1). Figure 2A shows CC_dFC_ = corr(sdFC, edFC) for the parameters (*K*, τ), while Figure 4B shows that the edFC calculated from the empirical BOLD signal. Figure 4C shows the sdFC matrix obtained by the larger CC_dFC_. In the lower row, we show the same analysis using the the hybrid rsSC matrix of the respective subject. Once again, in the context of dynamical functional connectivity, we find that the use of hybrid rsSC yields substantial improvement in the best fit between empirical and simulated BOLD activities (CC_dFC_(rsSC)≈0.72 while CC_dFC_(SC)≈0.29). In Figures 4G, H, we present the corresponding correlation analysis and scatterplots and conclusions as those found earlier (Figure 4). Here, we have presented the output for the same example subject (as the one in previous figures). However, this conclusion holds for all subjects similar to what we show in Figure 2, in Figures 4I, J, we show the respective statistical analysis and boxplots.

## 4 Discussion

In this study, we showed that a coupled Kuramoto oscillator system built on a novel brain connectome can yield simulated BOLD brain activities that strongly resemble actual BOLD signals observed during resting-state fMRI. We used the TVB computational platform with the Kuramoto model (Kuramoto, 2003) and generated simulated BOLD time series across a range of different model parameters (*K*, τ) (producing PSE colormaps like in Figure 2). This allowed us to optimize model parameters and tune generated synthetic BOLD signals that produce simulated functional connectivity (FC) most similar to actual observed FC. Overall, we found that there are important advantages in using hybrid rsSC as it can produce BOLD sequences and synthetic FC that follow the general trends of the empirical BOLD time series and empirical FC (Figures 2, 4).

Despite the fact that, in general, both sFC (simulated with rsSC/SC matrices) perform rather well in capturing the positive correlations observed in the empirical BOLD signals, only the rsSC ones can effectively produce negative correlations, closely matching those occurring in the empirical BOLD signals (Figure 3, see also Zhan et al., 2017).

Our study has a few limitations. First, we restricted ourselves to a specific frequency band during simulations, and thus, future studies should further explore different ranges of frequencies in the Kuramoto model, e.g., either in different Hz ranges or extracted directly from the empirical BOLD signals per node and per subject [see Lee and Frangou (2017); Popovych et al. (2021) and references therein]. Furthermore, one may validate these findings for different dynamical models or to consider additional relevant dynamical features such as noise or the use of neuroimaging data where the path lengths are also available. In the study by (Popovych et al., 2021), the authors compared BOLD simulated signals (with SC) obtained using different dynamical models, such as the Kuramoto phase oscillators and the Hopf limit-cycle oscillators. They reported that both models perform rather similarly and that the role of such a model is not crucial as well as differences in the quality of the simulated optimal BOLD signals when using different atlases (structural vs functional) and parcellations. In our study, we achieved a significantly better agreement between optimal sFC and eFC compared the ones reported in the literature (Popovych et al., 2021). Let us also stress that in this study, we do not seek to detect model parameter settings that could distinguish between carriers and non-carriers based on the presence or absence of the APOE ε4 gene or age and gender factors, which is a research direction we plan to take in the near future.

In summary, here, we showed that our recently proposed hybrid connectome rsSC can produce simulated synthetic BOLD signals that yield functional connectivity matrices, which are strikingly similar to those actually obtained during the resting state. Thus, we conclude by highlighting that existing publicly available open-source pipelines, such as the TVB platform, could be easily equipped to include an add-on module that incorporates rsSC for the neuroscientific community interested in the modeling of simulated fMRI BOLD time series.

## Data availability statement

The original contributions presented in the study are included in the article/Supplementary material, further inquiries can be directed to the corresponding author.

## Ethics statement

This study represents a secondary data analysis using imaging data obtained as part of a larger study. The latter one was carried out in accordance with guidelines set by the institutional review boards at the University of Wisconsin-Milwaukee and the Medical College of Wisconsin. The studies were conducted in accordance with the local legislation and institutional requirements. The participants provided their written informed consent to participate in this study.

## Author contributions

TM: Conceptualization, Formal analysis, Funding acquisition, Investigation, Methodology, Project administration, Resources, Software, Supervision, Validation, Visualization, Writing—original draft, Writing—review & editing. SD-P: Conceptualization, Methodology, Resources, Software, Writing—review & editing. IF: Data curation, Software, Writing—review & editing. ID: Data curation, Formal analysis, Software, Writing—review & editing. LZ: Data curation, Software, Writing—review & editing. AL: Conceptualization, Formal analysis, Funding acquisition, Methodology, Software, Supervision, Writing—review & editing.
