# Supplementary material for: Enhanced simulations of whole-brain dynamics using hybrid resting-state structural connectomes
Source: Front Comput Neurosci. 2023 Dec 19;17:1295395. doi: 10.3389/fncom.2023.1295395 (PMC10770256; doi:10.3389/fncom.2023.1295395)
Supplement: Supplementary file 1 [file Data_Sheet_1.pdf]

Supplementary Material

1 EMPIRICAL DATA

Structural and functional connectivity for 38 cognitively normal APOE $\varepsilon 4$ allele carriers aged 40–60 ($\mu = 50.8, \sigma = 0.99$) are compared with 38 age ($\mu = 50.9, \sigma = 0.99$) and sex-matched (16 male/22 female) non-carriers (control - non-carriers). Resting state functional MRI (rs-fMRI)—A T2*-weighted functional scan was obtained with an echo-planar pulse imaging (EPI) sequence (28 axial slices, 20×20 cm^2 FOV, 64×64 matrix, 3.125 mm $\times$ 3.125 mm $\times$ 4 mm voxels, TE = 40 ms, TR = 2,000 ms). The 8-minute rs-fMRI scan was acquired under a task-free condition (i.e., resting state): subjects were instructed to relax with eyes closed and to "not think about anything in particular". Imaging included T1-weighted MRI, resting state fMRI and diffusion weighted MRI. Freesurfer cortical parcellation and sub-cortical segmentation was performed to derive 80 regions-of-interest (ROIs) registered on the Desikan atlas (Desikan et al., 2006). The mean time-course was extracted from the pre-processed rs-fMRI data. Probabilistic tractography was used to create the structural connectome matrices, and normalized by the way-total of the corresponding seed ROIs. The detailed information on the imaging and processing steps can be found in (Korthauer et al., 2018).

2 SIGNED RESTING STATE STRUCTURAL CONNECTOME

In constructing a signed resting state structural connectome, we use a novel approach introduced in (Ajilore et al., 2013) and has already been used in several studies (see e.g. (Fortel et al., 2019, 2020, 2022, 2023) which takes into account both structural connectivity and functional time series to form a signed coupling interaction network or "signed resting state structural connectome" (signed rsSC) to describe neural excitation and inhibition. To this end, an energy representation of neural activity based on the Ising model from statistical mechanics which ultimately bypasses traditional BOLD correlations. The spin model is a function of a coupling interaction (with positive or negative values) and spin-states of paired brain regions. Observed functional time series represent brain states over time. A maximum pseudolikelihood with a constraint is used to estimate the coupling interaction. The constraint is introduced as a penalty function such that the learned interactions are scaled relative to structural connectivity; the sign of the interactions may infer inhibition or excitation over an underlying structure. The efficiency of this approach was validated in comparing a group of healthy APOE-$\varepsilon 4$ carriers (associated with genetic risk factor for Alzheimer's disease with a control (healthy) group of non APOE-$\varepsilon 4$ subjects.

Here, we briefly describe the computational aspect of this approach. First, we adapted the Ising model, a well-known spin-glass model from statistical physics in which the states, also referred to as "spin configurations", of interacting units – in our case brain regions connected by white matter edges – are constrained to be either 1 ("active") or -1 ("inactive"). As described in (Fortel et al., 2022; Tang et al., 2021) we construct a function-by-structure embedding (FSE) using a constrained pseudolikelihood estimation technique wherein pairwise interaction coefficients (represented as $(J_{i,j})$, with i and j representing ROIs in the brain network) are inferred from the observed data (BOLD time

series). As the model assumes binary data, we binarize the resting-state fMRI signals. The binarized activity pattern of all ROIs at time t ($t = 1, 2, \ldots, t_{\max}$) is ($\mathbf{s}(t) = s_1(t),\ s_2(t), \ldots s_N(t) \in \{-1, +1\}^N$).

The time series data was thresholded at zero (after global signal regression), yielding a binarized sequence of -1 or $+1$. Note that our procedure follows the same procedure as previously proposed by other groups in this general research area that leverages the Ising model (see (Fortel et al., 2022, 2023) for more details).

Note that $t_{\max}$ is determined as a result of the fMRI scan time. Here $(s_1(t) = \pm 1)$ indicates that an ROI is either active $(+1)$ or inactive (-1). First, the time series goes through a z-score normalization procedure, resulting in zero mean and unitary variance. The interaction $(J_{i,j})$ between two regions should be directly linked back to the diffusion MRI-derived structural connectivity between them as informed by tractography, so we add a constraint to the Hamiltonian function as:

$$H(s) = -\sum_{i<j} J_{i,j} s_i s_j, \tag{S1}$$

such that $|J_{i,j}| \propto W_{i,j}$, where $(W_{i,j})$ is the structural connectivity between pairs of ROIs, and the external force or bias terms are dropped in the case of resting-state. This ensures that in the pseudolikelihood estimation of $(\mathbf{J})$, we constrain it with the structural connectivity (under the assumption that structural connectivity informs spin models governing brain dynamics). Thus, the optimal interaction matrix $(\mathbf{J})$ is derived by maximizing the pseudo-likelihood function as:

$$\mathcal{L}_{\text{pseudo}}\left(\mathbf{J},\beta\right) = \prod_{t=1}^{t_{\max}} \prod_{i=1}^{k} \Pr\left(s_i(t) \middle| \mathbf{J},\beta, \mathbf{s}_{-i(t)}\right). \tag{S2}$$

Pseudolikelihood substitutes $\Pr(\mathbf{s})$ by the product of the conditional probabilities $\widetilde{p} = \Pr(s_i(t)|\mathbf{J}, \beta, \mathbf{s}_{-i}(t))$ observing one element $s_i(t)$ with all the other elements (denoted $\mathbf{s}_{-i}(t)$) fixed. To ensure that the magnitude of the coupling interactions is scaled relative to structural connectivity, the constraint is formulated as $|J_{i,j}| \approx \mu W_{i,j}$, where μ is a normalization constant and $W_{i,j}$ is the structural connectivity between ROI pairs. Without loss of generality, we assume that $\mu = 1$ with appropriate normalization. We therefore present a penalty-based optimization scheme to maximize the constrained log-pseudolikelihood function as:

$$\ell(\mathbf{J},\beta) = \frac{1}{t_{\max}} \ln\mathcal{L}_{\text{pseudo}}(\mathbf{J},\beta) - \frac{\lambda}{2} \sum_{i<j} (J_{i,j} - \text{sgn}(J_{i,j}) W_{i,j})^2. \tag{S3}$$

And the pseudolikelihood component expands as follows:

$$\begin{aligned} &\frac{1}{t_{\max}} \ln \mathcal{L}_{\text{pseudo}}\left(\mathbf{J},\beta\right) = \\ &= \frac{1}{t_{\max}} \sum_{t=1}^{t_{\max}} \sum_{i=1}^{N} \ln\left(\frac{\exp(\beta \sum_{k=1}^{N} J_{i,k} s_i(t) s_k(t))}{\exp(\beta \sum_{k=1}^{N} J_{i,k} s_k(t)) + \exp(-\beta \sum_{k=1}^{N} J_{i,k} s_k(t))}\right). \end{aligned} \tag{S4}$$

Our formulation here is based on the Boltzmann distribution under pseudolikelihood conditions. Thus, the numerator describes the energy of the system, while the denominator is the sum of all possible energies. Hence, there are only two terms in the denominator since $s_i(t)$ is binary (one positive, and one negative).

The likelihood function may be simplified by setting $C_i(t) = \beta \sum_{m=1}^{k} J_{i,m} s_m(t)$, resulting in:

$$\ell(\mathbf{J},\beta) = \frac{1}{t_{\text{max}}} \sum_{t=1}^{t_{\text{max}}} \sum_{i=1}^{N} C_i(t)s_i(t) - \ln(\exp(C_i(t)) + \exp(-C_i(t))) - \\ - \frac{\lambda}{2} \sum_{i<j} (J_{i,j} - \text{sgn}(J_{i,j}W_{i,j}))^2. \quad \text{(S5)}$$

Here we may construct the gradient ascent procedure with respect to $J_{i,j}$ by computing the partial derivative of the log-pseudolikelihood as:

$$\frac{\partial \ell}{\partial J_{i,j}} = \frac{1}{t_{\text{max}}} \sum_{t=1}^{t_{\text{max}}} \beta \{s_i(t)s_j(t) - s_j(t)\tanh(C_i(t))\} - \lambda(J_{i,j} - \text{sgn}(J_{i,j})W_{i,j}). \quad \text{(S6)}$$

The updating scheme follows:

$$J_{i,j}^{n+1} = J_{i,j}^{n} + \gamma \left. \frac{\partial \ell}{\partial J_{i,j}} \right|_n . \quad \text{(S7)}$$

Here, n is the iteration number and γ is the learning rate. In this way, the penalty function ensures that the inferred pairwise interaction is scaled relative to the estimated structure of the brain. This procedure is followed for all subjects in constructing an optimized **J** matrix per subject, which we term the resting-state structural connectome or rsSC.

In **Figure S1** we show the respective BOLD time series, In more detail, in **Figure S1**(A) we show the time evolution of the empirical BOLD signal. **Figure S1**(B) depicts the simulated BOLD signal with the parameters $(K, \tau) \approx (27, 20)$ found when optimizing CC_{FC} using the respective rsSC matrix while in **Figure S1**(C) we plot the respective simulated BOLD signal with the parameters $(K, \tau) \approx (39, 18)$ using the SC matrix. All BOLD signals in all three panels are scaled to range in $[-1, 1]$.

Analysis on the role of positive and negative coupling coefficients in rsSC vs SC connectommes in simulating high-fidelity fMRI correlations with a dynamical system

The inferred matrix rsSC with c_{ij} entries from the inverse Ising model (via a pseudo-likelihood maximization procedure that is further constrained by the structural connectivity, see (Fortel et al., 2022)) encodes information that reveals excitation vs inhibitory relationship between brain regions. Thus, by deploying the c_{ij} as coupling coefficients in a dynamical system (here coupled Kuramoto oscillators) we generate high-fidelity positive and negative fMRI correlations.

Our contribution is not in any way artificial, as the whole point of the paper is to outline and validate a Kuramoto-oscillator generative model with a novel brain connectome as the coupling coefficients for accurate simulations of resting-state fMRI dynamics. No prior attempt to our best knowledge has been able to match our reported accuracy in any artificial or non-artificial way.

Figure S3 depicts a visual comparison between the structural connectome (panel A), the c_{ij} matrix of the resting-state structural connectome (panel B), and functional connectome (panel C) presented in the manuscript. One can clearly observe that, at least visually, the rsSC matrix (panel B) actually looks more similar to the structural connectivity (panel A) than to the function connectivity (panel C).

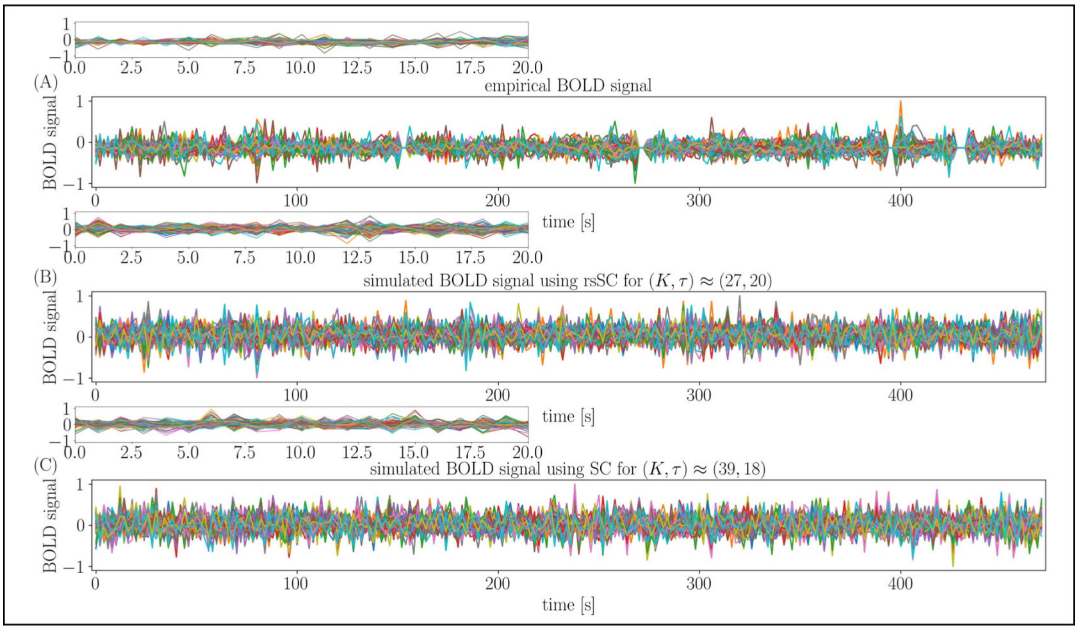

Figure S1. Bold signals. (A) Empirical BOLD signals. (B) Simulated BOLD signals with the parameters (K, τ) found when optimizing CC_{FC} using the respective rsSC matrix. (C) Simulated BOLD signals with the parameters (K, τ) found as in (B) but using the respective SC matrix. Note that all BOLD signals in all three panels are scaled to the range $[-1, 1]$. The small figures on top of the main panels show the respective zoomed areas for the first 20 seconds.

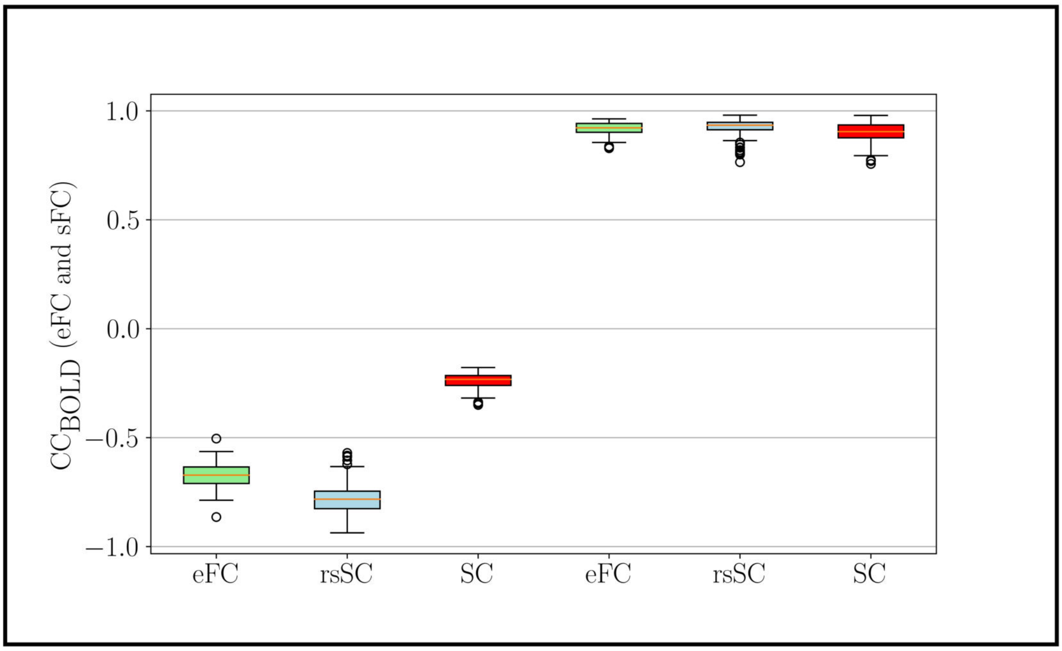

Figure S2. Statistical Analysis for negative and positive correlations in empirical and simulated FCs. Boxplots of the correlation coefficients for eFC and sFC obtained by using either SC or rsSC as the coupling coefficient matrices (non-carriers group) in the simulations. For each subject we considered the parameters (K, τ) which correspond to the 5 maximum values that optimize the similarity between eFC and sFC matrices (indicated with circles in **Figures 2 (A)**,**(D)**). The 3 leftmost boxplots indicate the negative correlations in the actual eFC (light green), simulated using rsSC (light blue) and using standard structural connectome SC (red).

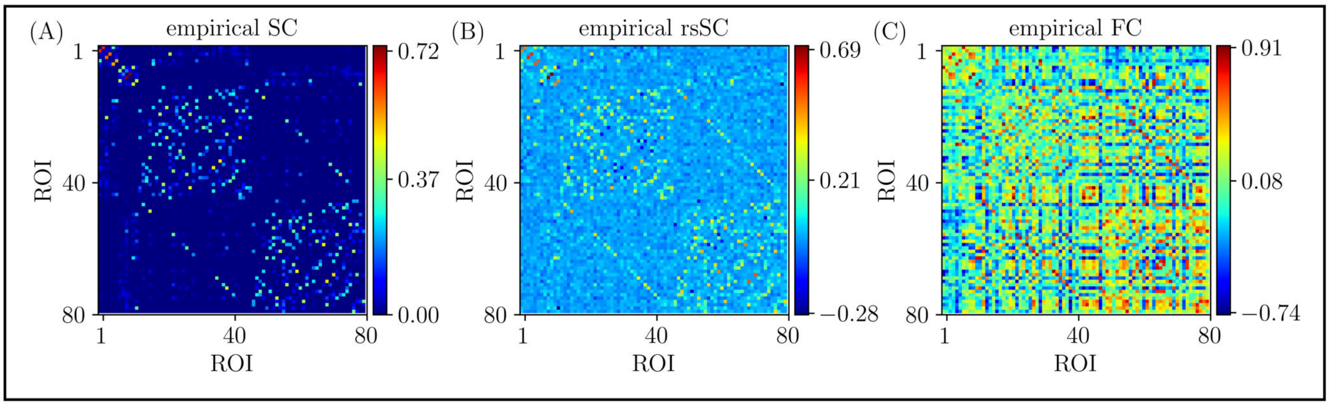

Figure S3. A visual comparison. Structural connectome (A), resting-state structural connectome (B) and functional connectome (C).

In the **Figure** S4, we show the Pearson correlation coefficient boxplots between empirical (i) SC and rsSC, (ii) SC and FC and (iii) rsSC and FC matrices for all non-carriers (panle A) and carriers (panel B) datasets. Out of the three comparisons, the SC and rsSC connectomes are most similar as their Pearson correlation coefficient indicates, while the SC and FC connectomes are the least similar. The rsSC and FC connectomes exhibit intermediate similarity with a Pearson correlation coefficient ≈ 0.6. If simply going by the value of the correlation, then one must (also by mistake) argue that the rsSC artificially captures mostly the structural connectivity information.

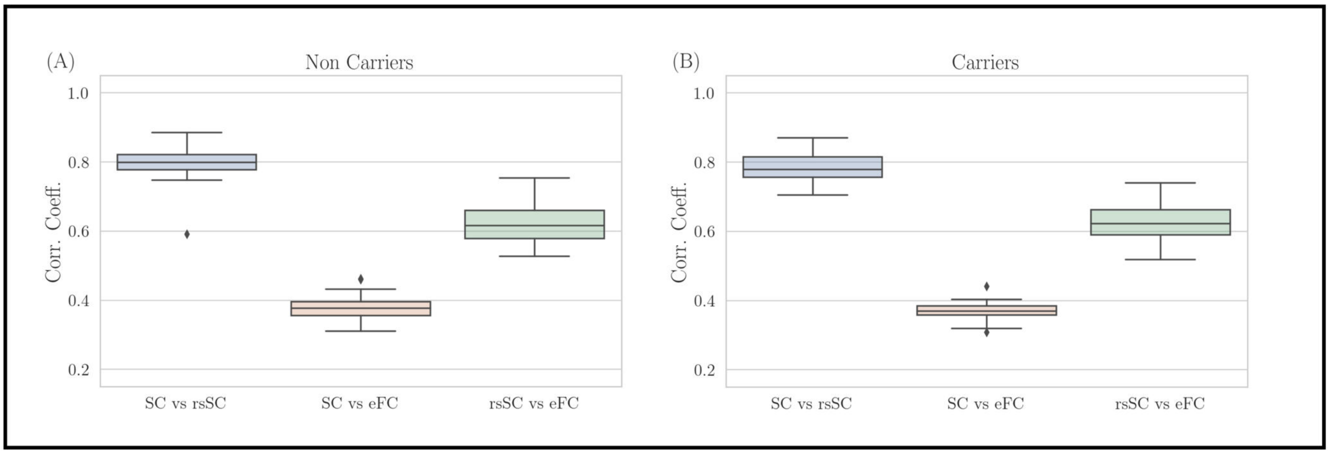

Figure S4. Pearson correlation coefficient boxplots. Empirical (i) SC and rsSC, (ii) SC and FC and (iii) rsSC and FC matrices for all (A) non-carriers and (B) carriers datasets.

Next, we also studied what happens when some random weights of the SC are made negative, to check whether the presence of negative weights alone allows for more expressive dynamical regimes.

SC matrices

To this end, we modified the SC matrices as follows: We used the same original SC from the non-carrier subject in **Figures 1-4** of the main manuscript. Then 10% of the positive connections were selected randomly and their signs were reversed to negative (all connections in the original SC matrix are non-negative). This produced the first matrix. Similarly, we repeated the same procedure to randomly select and sign-flip an additional 20% of the non-zero positive connections to produce the second matrix. In total, this produced the 10%, 30%, 50%, 70% and 90% sign-reversed SC matrices **Figures** S5 and S6 shows the result for 10% and 30% respectively.

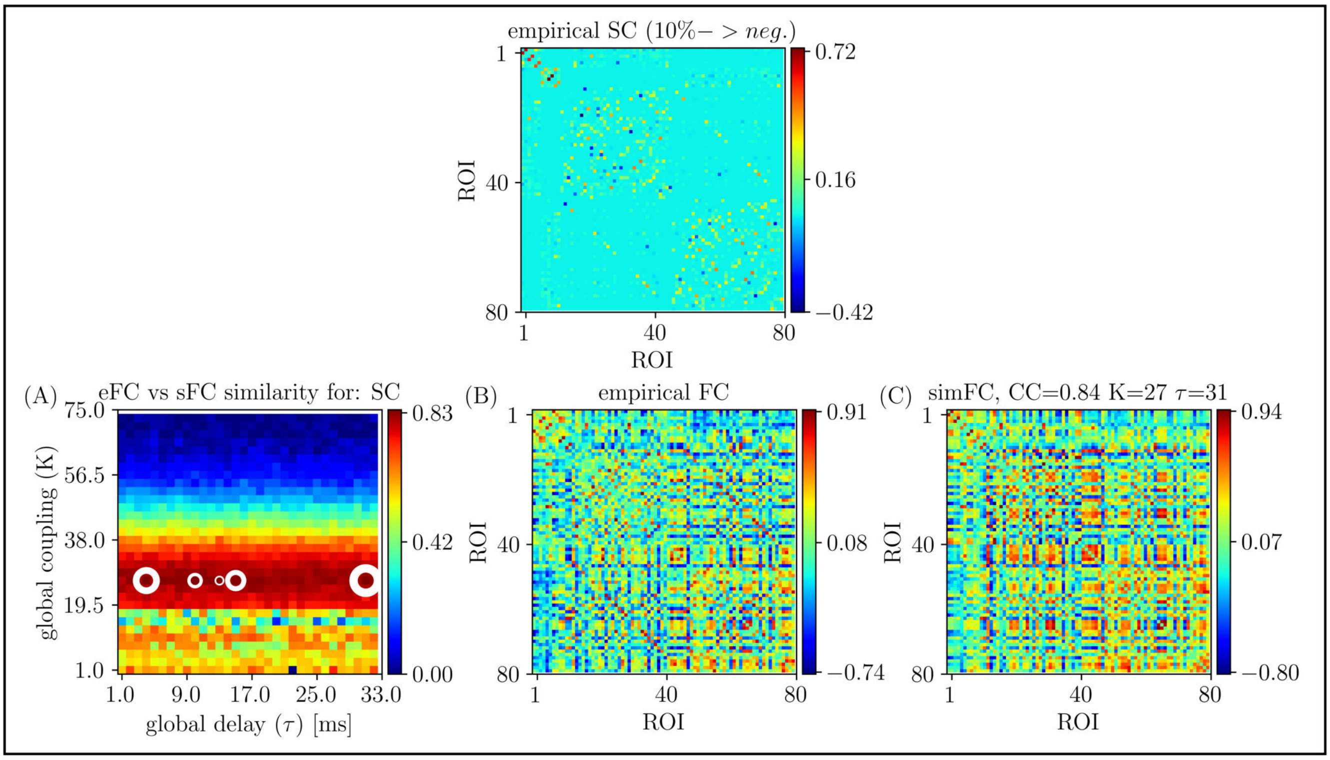

Figure S5. A SC matrix with 10% random negative entries. Upper row: we selected 10% of the positive connections randomly and reversed their signs to negative. All connections in the original SC matrix are non-negative. Bottom row: parameter sweep exploration performing a similar analysis to the one presented in **Figures 2** & **4** of the manuscript.

Indeed, with a low fraction (10%) of sign-reversal the agreement between empirical FC and simulated FC (after fitting our proposed Kuramoto model) improved, suggesting that the presence of negative-valued coupling in the Kuramoto model is indeed necessary to sufficiently recover negative correlations in the functional connectivity. However, as expected with higher-fraction (50%, 70% and 90%, not shown here) sign-reversals of SC the agreement between empirical and simulated FC decreased again, supporting the notion of an optimal Excitation-Inhibition balance as we argue in this manuscript.

rsSC matrices

We have also performed a similar experiment by modifying the rsSC matrix of the same representative subject as follows: We first calculated the total amount of negative connections in rsSC. Then, we

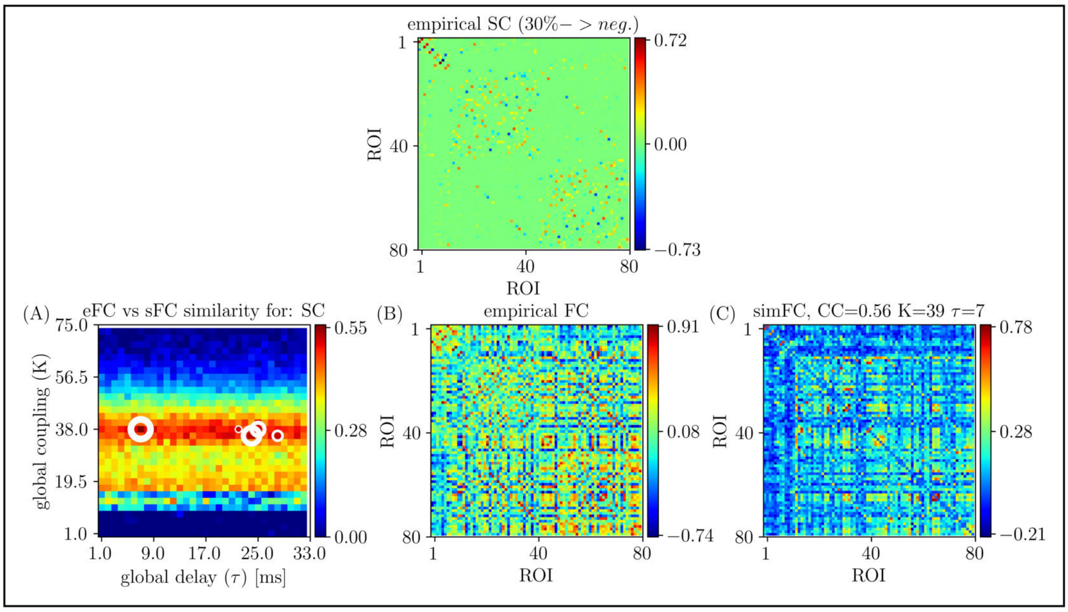

Figure S6. A SC matrix with 30% random negative entries. Upper row: we selected 30% of the positive connections randomly and reversed their signs to negative. All connections in the original SC matrix are non-negative. Bottom row: parameter sweep exploration performing a similar analysis to the one presented in **Figures 2** & **4** of the manuscript.

randomly selected 10% of these negative connections and turned them into positive by taking their absolute values. This produced the first sign-flipped rsSC matrix. We repeated the same process as before, yielding a total of 5 sign-flipped rsSC matrices at various fractions (10%, 30%, 50%, 70% and 90%) with randomly reversed signs from negative to positive. The general trend of the rsSC findings here is complementary to those in the previous SC sign-reversal experiments. Here, as the rsSC sign-reversal percentage (negative to positive) increased the agreement between simulated and empirical FC matrices worsened. **Figures** S7 and S8 show the result for 10% and 30% respectively.

Taken together, given that structural connectivity is always non-negative, our simulations demonstrated that with structural connectivity as the coupling coefficients of a Kuramoto-oscillator model the recovery of negative correlations in FC is suboptimal even with the consideration of global delay. Thus, the main contribution of our manuscript is a principled biophysically-informed generative framework that leads to a signed coupling matrix (i.e. the rsSC) capable of generating substantially more accurate functional connectivity. Indeed, our model exhibits more expressive dynamical regimes and hence a better fit with the data.

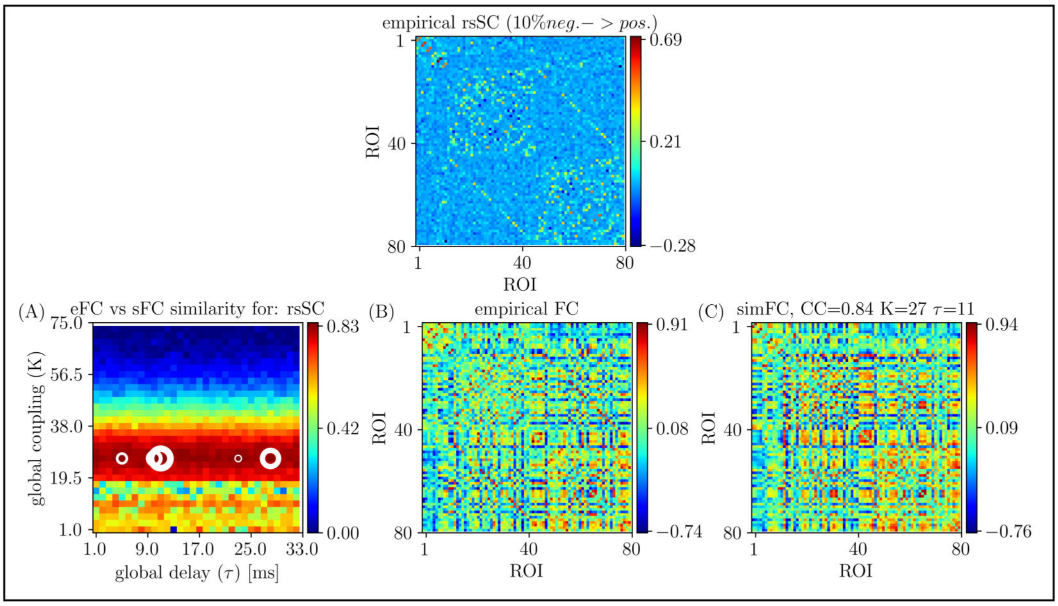

Figure S7. A rsSC matrix with 10% random negative entries Upper row: After having calculated the total amount of negative connections in rsSC, we randomly selected 10% of these negative connections and turned them into positive by taking their absolute values. Bottom row: parameter sweep exploration performing a similar analysis to the one presented in **Figures 2** & **4** of the manuscript.

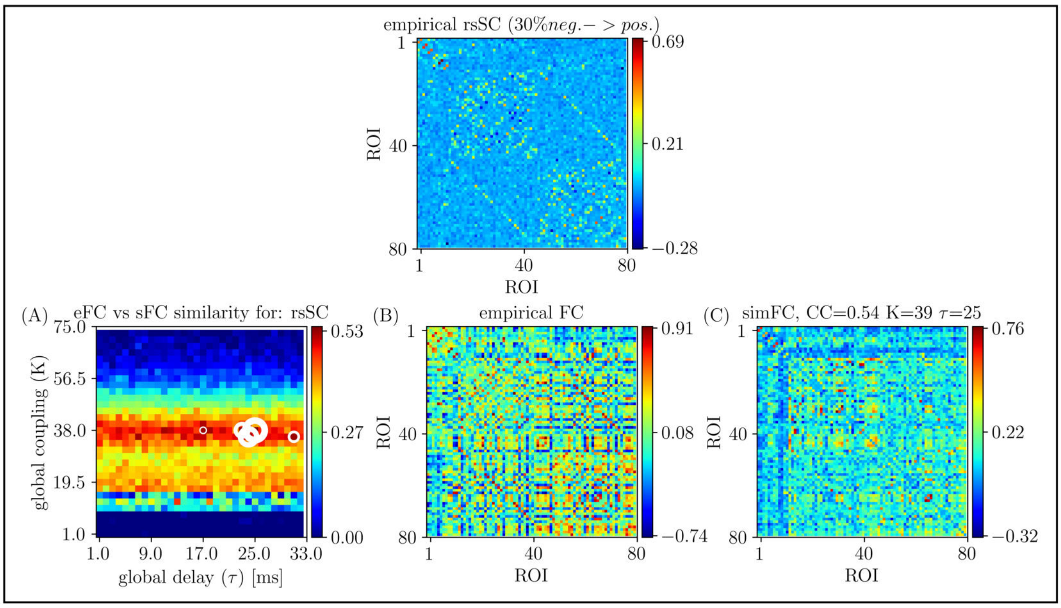

Figure S8. A rsSC matrix with 30% random negative entries Upper row: After having calculated the total amount of negative connections in rsSC, we randomly selected 30% of these negative connections and turned them into positive by taking their absolute values. Bottom row: parameter sweep exploration performing a similar analysis to the one presented in **Figures 2** & **4** of the manuscript.

REFERENCES

Ajilore, O., Zhan, L., GadElkarim, J., Zhang, A., Feusner, J., Yang, S., et al. (2013). Constructing the resting state structural connectome. *Frontiers in Neuroinformatics* 7. doi:10.3389/fninf.2013.00030

Desikan, R. S., Ségonne, F., Fischl, B., Quinn, B. T., Dickerson, B. C., Blacker, D., et al. (2006). An automated labeling system for subdividing the human cerebral cortex on mri scans into gyral based regions of interest. *NeuroImage* 31, 968–980. doi:https://doi.org/10.1016/j.neuroimage.2006.01.021

Fortel, I., Butler, M., Korthauer, L. E., Zhan, L., Ajilore, O., Driscoll, I., et al. (2019). Brain dynamics through the lens of statistical mechanics by unifying structure and function. In *Medical Image Computing and Computer Assisted Intervention – MICCAI 2019*, eds. D. Shen, T. Liu, T. M. Peters, L. H. Staib, S. Essert, Carolineand Zhou, P.-T. Yap, and A. Khan (Cham: Springer International Publishing), 503–511

Fortel, I., Butler, M., Korthauer, L. E., Zhan, L., Ajilore, O., Sidiropoulos, A., et al. (2022). Inferring excitation-inhibition dynamics using a maximum entropy model unifying brain structure and function. *Network Neuroscience* 6, 420–444. doi:10.1162/netn_a_00220

Fortel, I., Korthauer, L. E., Morrissey, Z., Zhan, L., Ajilore, O., Wolfson, O., et al. (2020). Connectome Signatures of Hyperexcitation in Cognitively Intact Middle-Aged Female APOE-ε4 Carriers. *Cerebral Cortex* 30, 6350–6362. doi:10.1093/cercor/bhaa190

Fortel, I., Zhan, L., Ajilore, O., Wu, Y., Mackin, S., and Leow, A. (2023). Disrupted Excitation-Inhibition balance in cognitively normal individuals at risk of alzheimer's disease. *J Alzheimers Dis* 95, 1449–1467. doi:10.3233/JAD-230035.PMID:37718795

Korthauer, L., Zhan, L., Ajilore, O., Leow, A., and Driscoll, I. (2018). Disrupted topology of the resting state structural connectome in middle-aged apoe ε4 carriers. *NeuroImage* 178, 295–305. doi:https://doi.org/10.1016/j.neuroimage.2018.05.052

Tang, H., Ma, G., He, L., Huang, H., and Zhan, L. (2021). Commpool: An interpretable graph pooling framework for hierarchical graph representation learning. *Neural Networks* 143, 669–677. doi:https://doi.org/10.1016/j.neunet.2021.07.028
